# Lymph Node Involvement in Oesophageal Carcinoma: A Single-Centre Observational Study From Western India

**DOI:** 10.7759/cureus.17741

**Published:** 2021-09-05

**Authors:** Ajay K Boralkar, Abdul Rafe, Bhushan Bhalgat

**Affiliations:** 1 Department of Surgery, Government Cancer Hospital, Aurangabad, IND

**Keywords:** esophagus adenocarcinoma, abdominal lymph nodes, squamous cell carcinoma esophagus, adenocarcinoma esophagus, thoracic lymph nodes, skip node metastases

## Abstract

Introduction

Lymph node involvement is the most important predictor of prognosis in oesophageal cancer. The present study describes our single-centre experience of lymphadenopathy in oesophageal carcinoma cases at a tertiary care centre in the Marathwada region of Maharashtra state in western India.

Methods

This descriptive study included 31 patients who were operated for oesophageal carcinoma at the State Cancer Hospital in Marathwada from August 2015 to September 2017. Thirty patients underwent three-field lymph node dissections, and one patient underwent Ivor Lewis surgery with two-field lymph node dissections. Three-field lymph node dissections were through a thoracotomy, followed by laparotomy and left cervical incision. The lymphatic metastases were categorised as (a) adjacent node metastases, (b) multiple levels of lymph node metastases, and (c) skip node metastases. The histopathological assessment of the removed specimen and lymph nodes was done. Pathologists evaluated the character and depth of the primary tumour and its invasion and node involvement. The location and numbers of positive and negative nodes were recorded.

Results

A total of 31 patients were included in the study, of which 17 had lymph node involvement. A total of 946 lymph nodes were dissected and examined, and the average number of lymph nodes removed per patient was 30.51. Among the 28 squamous cell carcinoma cases, lymph node involvement was found in 14 cases (50%) whereas, in adenocarcinoma, all the three cases showed lymph node involvement. In 11 cases of squamous cell carcinoma, thoracic lymph nodes were involved, whereas abdominal lymph nodes were involved in nine and cervical lymph nodes in two cases. Thoracic lymph nodes were involved in two cases of adenocarcinoma and abdominal lymph nodes were involved in one case of adenocarcinoma.

Conclusions

Squamous cell carcinoma was the predominant type, and lymph node metastasis was observed in 50% of these cases. Thoracic lymph nodes were more commonly involved. Tumour staging T2 and T3 had an increasing percentage of lymph nodes involved. Lymph node involvement was more in moderately differentiated and undifferentiated oesophageal cancers.

## Introduction

Oesophageal cancer is among the top three gastrointestinal malignancies in our country, and it has a poor prognosis. Lymphatic drainage of oesophagus runs along the oesophageal axis vertically and drains into cervical and abdominal lymph nodes. There is widespread lymph node metastasis observed in oesophageal cancer. The lymph node spread of oesophageal cancer is varied and correlates with T status, stage, grading, perineural invasion, length and width of the primary tumour and histopathology of the tumour. The status of lymph node involvement is the most important prognostic marker in oesophageal cancer [1-4]. Postoperatively, the proportion of affected lymph nodes removed is an important factor that impacts the outcome of the disease. The clinical and histological presentation of the disease varies with the geographic region. An in-depth understanding of the lymph node involvement in oesophageal cancers can be of great help for treating oncology professionals [5-7]. The present study describes our single-centre experience of lymphadenopathy in oesophageal carcinoma at a tertiary care centre in the Marathwada region of Maharashtra state in western India.

## Materials and methods

This descriptive study included 31 patients who were operated for oesophageal carcinoma at the State Cancer Hospital in Marathwada from August 2015 to September 2017. The Institutional Ethics Committee approved the study protocol. All patients diagnosed with carcinoma of the oesophagus, with surgery being their primary modality of treatment, were included after their informed consent for study participation. Exclusion criteria were metastasis to viscera, medically unfit or not willing for surgery. After collecting the preliminary data, a thorough history was taken in each case. A thorough general and physical examination was carried out for each patient to determine any associated disease and judge fitness for general anaesthesia and surgery.

All routine investigations were carried out. Endoscopy and endoscopic biopsy were done to confirm the pathology. Radiological investigations such as chest x-ray, ultrasonography of the neck and the abdomen and contrast study of the neck, thorax and abdomen were done. Each patient was discussed in a multi-disciplinary team meeting and a plan of treatment was decided.

Thirty patients underwent total transthoracic oesophagectomy with three-field lymph node dissection, and one patient underwent Ivor Lewis surgery with two-field lymph node dissections. Thus, 30 patients underwent three-field lymph node dissections, and one underwent two-field lymph node dissection. Three-field lymph node dissections were through a thoracotomy, followed by laparotomy and left cervical incision. A greater curvature gastric tube was pulled to the neck in 30 cases and thorax in 1 case for esophagogastric anastomosis. Right fourth intercostal thoracotomy was performed. Then the oesophagus with the tumour was removed along with adjoining tissues, i.e., both pleural surfaces, the pericardium and all the lympho-vascular tissues between the spine and oesophagus. The thoracic lymphatic duct was resected throughout its course in the posterior mediastinum, and so a complete dissection of the middle and lower mediastinal nodes, including the peri-oesophageal, para-hiatal, subcarinal and aortopulmonary window nodes, was done. The upper abdominal and retroperitoneal lymph nodes were removed. The left cervical incision was used to clear the superior mediastinal and the cervical lymph nodes. The nodes along the mediastinal course of recurrent laryngeal nerves, para-oesophageal nodes in the upper thorax, pretracheal nodes, tracheobronchial nodes, the lower cervical deep nodes postero-lateral to the carotid sheath and the supraclavicular nodes were removed. Lymph nodes >1 cm on the short-axis diameter were considered significant although all stations were dissected and sent for histopathological examination.

Lymph node mapping

Lymphatic nodes were named as per the Japanese Esophageal Society guidelines for oesophageal diseases and divided into (a) cervical lymph nodes, (b) thoracic lymph nodes, and (c) abdominal lymph nodes [8].

Two-field lymph node dissection involved the resection of lymph nodes within the mediastinal and abdominal lymph node stations. Right thoracotomy was done and the paraesophageal, bilateral intrathoracic recurrent laryngeal nerve chain nodes, subaortic arch nodes, subcarinal nodes, and bilateral pulmonary hilar nodes were dissected. The paracardial, celiac nodes, nodes along the left gastric artery, and common hepatic artery nodes were dissected through an upper midline laparotomy. Cervical collar incision was made and the cervical recurrent laryngeal nerve chain nodes, internal jugular nodes below the level of the cricoid cartilage, supraclavicular nodes, deep cervical nodes, and cervical para oesophageal nodes were dissected bilaterally.

The location of oesophageal carcinoma was described as follows: (a) cervical oesophagus, 15-20 cm from incisors; (b) upper thoracic oesophagus, >20-25 cm from incisors; (c) middle thoracic oesophagus, >25-30 cm from incisors; (d) lower thoracic oesophagus, >30-40 cm from incisors.

The pattern and extent of lymphatic spread was described as follows: (a) adjacent node metastases, same segment of the oesophageal lesion; (b) multiple levels of lymph node metastases, adjacent and other nodes; (c) skip node metastases in which adjacent nodes are uninvolved, but other levels are involved.

Histopathological assessment of the removed specimen and lymph nodes

Each group was assigned its localisation; they were fixed in 10% formalin, embedded in paraffin, stained with haematoxylin and eosin and examined by light microscopy. Pathologists evaluated the character and depth of primary tumour and its invasion and node involvement. The positive and negative node numbers and their locations were recorded. Postoperatively, the patient was kept electively intubated for one day and in ICU for four to five days and afterwards in the general ward. The hospital stay was 10-12 days. However, in the case of a postoperative complication, the hospital stay was prolonged.

## Results

Among the 31 patients studied, lymph node involvement was found in 17 patients. The most common symptoms were dysphagia and weight loss.

Out of 31 cases, 15 cases were given neoadjuvant chemotherapy before surgery, whereas 16 cases underwent surgery directly. Tables 1-4 describe the characteristics of the cases concerning age, sex, histopathology, staging and lymph node metastasis.

**Table 1:** Distribution of age and sex with histopathology of the primary lesion

Histopathology	41-50 years		51-60 years		61-70 years		71-80 years	
	Male	Female	Male	Female	Male	Female	Male	Female
Squamous cell carcinoma	5 (16.1%)	5 (16.1%)	5 (16.1%)	5 (16.1%)	4 (12.9%)	2 (6.5%)	1 (3.2%)	1 (3.2%)
Adenocarcinoma	1 (3.2%)	1 (3.2%)	1 (3.2%)	0	0	0	0	0

**Table 2:** Distribution of the location of the primary lesion with histopathology of the primary lesion

Histopathology	Upper thoracic oesophagus	Mid thoracic oesophagus	Lower thoracic oesophagus	Total
Squamous cell carcinoma	4 (12.9%)	8 (25.6%)	16 (51.5%)	28 (90.4%)
Adenocarcinoma	1 (3.2%)	0	2 (6.5%)	3 (9.6%)

**Table 3:** Distribution of the number of cases with the stage of the disease

Stage	Number of cases
1a	0
1b	6 (19.3%)
2a	6 (19.3%)
2b	1 (3.2%)
3a	5 (16.1%)
3b	10 (32.2%)
4a	3 (9.6%)
4b	0
Total	31

**Table 4:** Distribution of cases of lymph node metastases with primary lesion stage

Stage of the primary lesion (n)	Patients with cervical lymph node involvement	Patients with thoracic lymph node involvement	Patients with abdominal lymph node involvement	Patients with overall lymph node involvement
T1 (5)	0	1	1	2 (40%)
T2 (20)	2	8	7	10 (50%)
T3 (6)	0	4	2	5 (83%)
Total	2	13	10	17

Table 5 shows that maximum primary lesions with lymph node involvement were seen in lower thoracic oesophagus and lymphatic spread towards thoracic lymph nodes. Among the 28 squamous cell carcinoma cases, lymph node involvement was found in 14 cases (50%) whereas, in adenocarcinoma, all the three cases showed lymph node involvement. In 11 cases of squamous cell carcinoma, thoracic lymph nodes were involved, whereas abdominal lymph nodes were involved in nine cases and cervical lymph nodes in two cases. Thoracic lymph nodes were involved in two cases of adenocarcinoma. Abdominal lymph nodes were involved in one case of adenocarcinoma.

**Table 5:** Distribution of lymph nodes with the primary lesion location

Location of the primary lesion	Patients with cervical lymph node involvement	Patients with thoracic lymph node involvement	Patients with abdominal lymph node involvement	Patients with overall lymph node involvement
Upper thoracic (5)	1	1	2	2
Mid thoracic (8)	0	4	3	5
Lower thoracic (18)	1	8	5	10
Total	2	13	10	17

Table 6 shows that maximum lymph node involvement occurred in G2 and maximally to thoracic lymph nodes.

**Table 6:** Distribution of lymph nodes with the primary lesion grade

Grade of the primary lesion	Patients with cervical lymph node involvement	Patients with thoracic lymph node involvement	Patients with abdominal lymph node involvement	Patients with overall lymph node involvement
G1 - well differentiated (10)	0	3	1	4 (40%)
G2 - moderately differentiated (20)	2	10	8	12 (60%)
G3 - poorly differentiated (1)	0	0	1	1 (100%)
Total (31)	2	13	10	17 (55%)

Table 7 shows the distribution of the length of the primary lesion with lymph node involvement; maximum lymph node involvement was noticed in 41- to 60-mm length tumours.

**Table 7:** Distribution of lymph nodes with the primary lesion length

Length of the primary lesion	Patients with cervical lymph node involvement	Patients with thoracic lymph node involvement	Patients with abdominal lymph node involvement	Patients with overall lymph node involvement
Up to 40 mm (5)	0	0	1	1 (20%)
More than 40 mm (26)	2	13	9	16 (61.5%)
Total (31)	2	13	10	17 (54.8%)
Mean length	64 mm	61.5 mm	48.6 mm	55.32 mm

Table 8 shows the distribution of the width of the primary lesion with lymph node involvement; 6- to 10-mm width tumours had maximum lymph node involvement. Overall, the size of the tumour was not indicative of lymph node involvement.

**Table 8:** Distribution of lymph nodes with the primary lesion width

Width of the primary lesion	Patients with cervical lymph node involvement	Patients with thoracic lymph node involvement	Patients with abdominal lymph node involvement	Patients with overall lymph node involvement
Up to 10 mm (18)	2	8	6	10 (55.6%)
More than 10 mm (13)	0	5	4	7 (53.8%)
Total (31)	2	13	10	17
Mean width	8.0 mm	11.6 mm	10.9 mm	11.03 mm

Lymphovascular and perineural invasion of the primary lesion was observed in seven cases. Of these, in six cases, the primary lesion was in the lower thoracic oesophagus, and the primary lesion was in the mid thoracic oesophagus in one case (Table 9).

**Table 9:** Distribution of the location of the primary tumour with lymphovascular and perineural invasion of the primary lesion

Location of the primary tumour	Cervical oesophagus	Upper thoracic oesophagus	Mid thoracic oesophagus	Lower thoracic oesophagus	Total
Lymphovascular and perineural invasion of the primary lesion					
Present	0	0	1 (3.2%)	6 (19.4%)	7 (22.6%)
Absent	0	5 (16.1%)	6 (19.4%)	13 (41.9%)	24 (77.4%)
Total	0	5 (16.1%)	7 (22.6%)	19 (61.3%)	31 (100%)

Among squamous cell carcinoma cases, multiple lymph node involvements were observed in two and skip lymph node involvement (directly in the abdomen) in one out of eight cases of the middle thoracic oesophagus. Directly skip nodes in the abdomen were seen in two out of 18 cases of lower thoracic oesophagus carcinoma. Skip nodes to cervical lymph nodes were not seen. Multiple lymph node involvements were observed in two out of 18 cases of lower thoracic oesophagus carcinoma. In the two lower thoracic oesophageal adenocarcinoma cases, only thoracic lymph nodes were involved. Abdominal skip lymph nodes were observed in the single case of upper thoracic oesophageal adenocarcinoma. Table 10 shows the distribution of lymph nodes with lymphovascular and perineural invasion of the primary lesion. 

**Table 10:** Distribution of lymph nodes with lymphovascular and perineural invasion of the primary lesion

Lymph nodes dissected	Patients with cervical lymph node involvement	Patients with thoracic lymph node involvement	Patients with abdominal lymph node involvement	Patients with overall lymph node involvement
Lymphovascular and perineural invasion of the primary lesion				
Present (7)	0	3	3	5
Absent (24)	2	10	7	12
Total	2	13	10	17

On analysis of cases with positive cervical lymph nodes, intraoperative assessment revealed more lymph node involvement as compared to the CT and histopathology findings. On CT with contrast, no cervical lymph node involvement was noticed, but 22.6% of cases showed cervical lymph node involvement intraoperatively, and 6.5% of cases showed cervical lymph node involvement on histopathology. Similarly, in the cases of involvement of thoracic lymph nodes, intraoperative findings revealed more lymph node involvement as compared to contrast CT. In the case of thoracic lymph nodes, 32.2% involvement was found on CT, 51.6% intraoperatively and 41.9% on histopathology. In the case of intra-abdominal lymph nodes, CT showed involvement in only 3.2% cases; intraoperatively 19.6% cases showed involvement and 32.6% cases were proved to be involved on histopathology. The accuracy of CT with histopathology for cervical lymph nodes was 0%, for thoracic lymph nodes was 76.9% and for abdominal lymph nodes was 30%. The average accuracy of CT with histopathology was 52% (Table 11).

**Table 11:** Distribution of the pattern of lymph node involvement with contrast CT, intraoperative and histopathological findings

Lymph nodes dissected	Cervical lymph nodes	Thoracic lymph nodes	Abdominal lymph nodes	Total
Contrast CT findings	0	10 (32.3%)	3 (9.7%)	13 (41.9%)
Intraoperative findings	7 (22.6%)	16 (51.6%)	6 (19.4%)	29 (93.5%)
Histopathological findings	2 (6.5%)	13 (41.9%)	10 (32.2%)	25 (80.6%)
Accuracy of CT with histopathological findings	0%	76.9%	30%	52%

In our study, complications were observed in four cases. There was recurrent laryngeal nerve injury in two cases, splenic artery injury in one case and thoracic lymphorrhea in one case.

**Figure 1:**
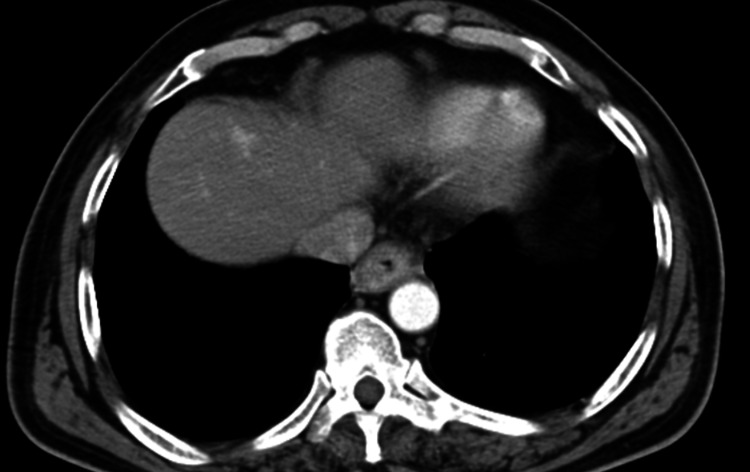
CT image of oesophageal carcinoma in the lower third region

**Figure 2:**
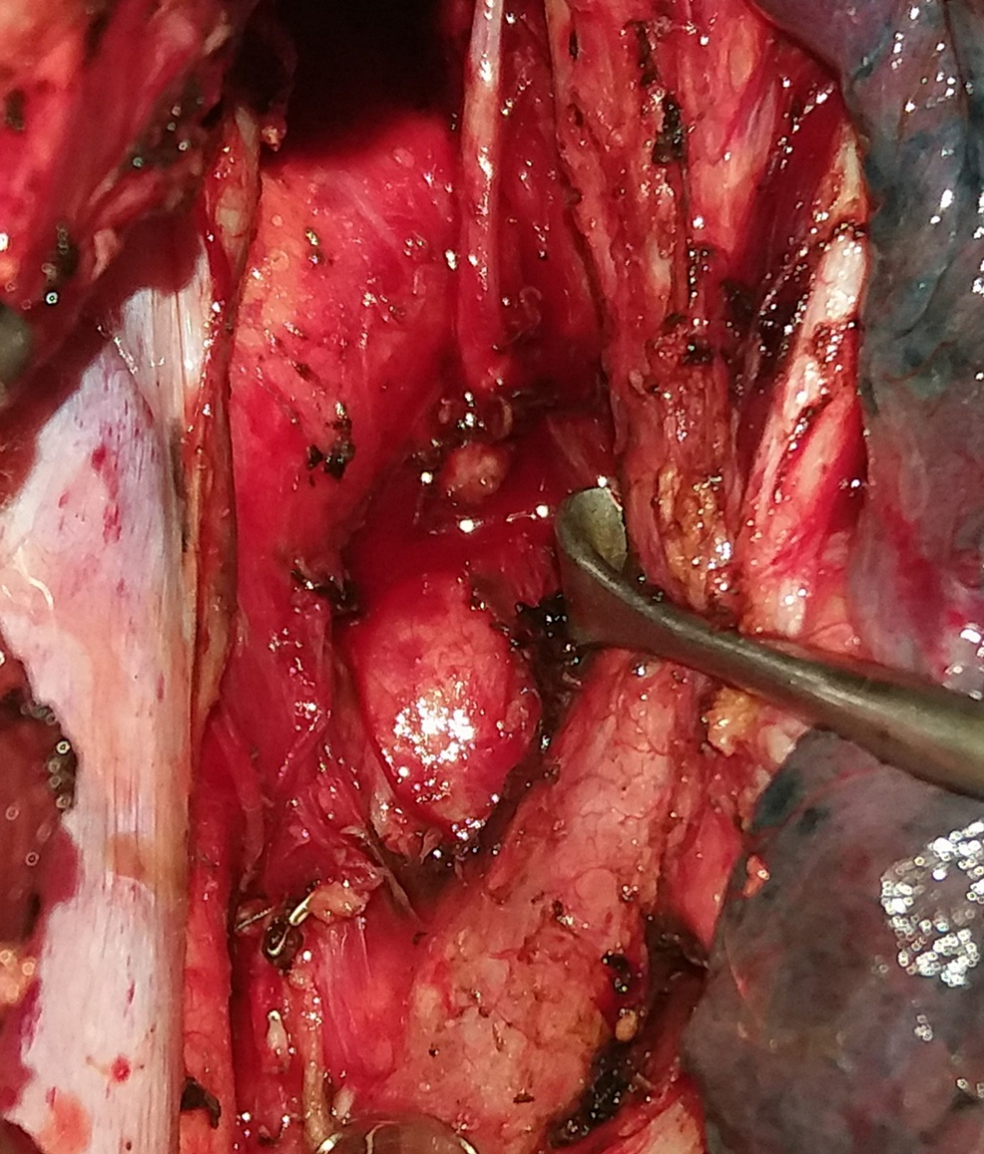
Aorto-pulmonary lymph nodes

Figure 1 shows the CT image of the carcinoma of the lower third of oesophagus. Figure 2 shows the aorto-pulmonary node dissection.

## Discussion

In this study, 17 out of 31 patients were found to have lymph node involvement. The average number of lymph nodes removed per patient was 30.51. The lymph node counts are lower than reported in the literature. Kato et al. reported 74.9 (mean) nodes dissected and Nishihara et al. reported an average of 82 lymph nodes dissected in oesophageal carcinoma surgeries [9,10]. In an Indian study done on cadavers, a very high average number of lymph nodes (183.6) relevant to the three-field lymphadenectomy for oesophageal carcinoma was reported. The authors cited the complexity of lymph node dissection (functional lymphadenectomy) due to vital structures in proximity in oesophageal carcinoma surgery as the reason for yielding less number of nodes in surgeries as compared to higher numbers yielded in the cadaveric study [11]. Akutsu et al. have highlighted the importance of metastatic lymph node number as the best predictor of overall survival [12]. In our study results, T2 and T3 and moderately differentiated or undifferentiated tumours showed more lymph node involvement. Sgourakis et al. found that deeper submucosal layer invasion and microvascular invasion were the predictors of a positive lymph node in squamous cell carcinoma of oesophagus [13]. In adenocarcinoma, the lymphovascular invasion was the best predictor. Hagens et al. reported that squamous cell carcinoma, as well as adenocarcinoma, metastasizes to lymph node stations in cervical, thoracic, and abdominal regions, irrespective of the location of the primary tumour [14]. Matsuda et al. reviewed three-field dissection for carcinoma oesophageal carcinoma, stating that there is a high incidence of lymph node metastasis in this cancer [15]. They put forth the importance of optimal lymph node dissection as there needs to be a balance between the benefit of extended lymph node dissection and increased postoperative complications associated with it. Similar to our observations, Berry mentioned that CT is limited in giving the local extent and lymph node involvement status [16].

There is a need for studies on the subject. Currently, an international research project is being carried out (TIGER study) involving oesophageal cancer institutes across the world to assess the lymph node status in carcinoma of oesophagus. This study aims to provide details for uniform classification and staging, which can help in designing tailor-made and patient-centred treatment options [17].

Thus, in oesophageal carcinoma, lymphatic metastasis may develop by three routes: longitudinally along the submucosa plexus to regional and non-regional lymph nodes, perpendicularly through the muscularis propria to regional lymph nodes and to the thoracic duct and systemic venous plexus. Therefore, it might be logical to interpret that not only the mediastinal lymph nodes but also the cervical and upper abdominal group of lymph nodes are part of regional lymphatic drainage of oesophagus.

Limitations

This study is descriptive and single centre based, and the sample size was small. Follow-up is another limitation, and it does not provide the details of outcomes over a long period.

## Conclusions

Squamous cell carcinoma was the predominant type and lymph node metastasis was found in 50% cases. Lymph node involvement was more in moderately differentiated and undifferentiated oesophageal cancers. The utility of CT was found to be limited in diagnosing lymph node involvement. The varied presentation of the disease with respect to lymphatic spread and the prognostic impact should encourage further research on the topic so as to better understand and manage oesophageal cancer.
